# Sexually Dimorphic Effects of Catechol-O-Methyltransferase (COMT) Inhibition on Dopamine Metabolism in Multiple Brain Regions

**DOI:** 10.1371/journal.pone.0061839

**Published:** 2013-04-16

**Authors:** Linda M. Laatikainen, Trevor Sharp, Paul J. Harrison, Elizabeth M. Tunbridge

**Affiliations:** 1 Department of Psychiatry, University of Oxford, Oxford, United Kingdom; 2 Department of Pharmacology, University of Oxford, Oxford, United Kingdom; University of Queensland, Australia

## Abstract

The catechol-O-methyltransferase (COMT) enzyme metabolises catecholamines. COMT inhibitors are licensed for the adjunctive treatment of Parkinson's disease and are attractive therapeutic candidates for other neuropsychiatric conditions. COMT regulates dopamine levels in the prefrontal cortex (PFC) but plays a lesser role in the striatum. However, its significance in other brain regions is largely unknown, despite its links with a broad range of behavioural phenotypes hinting at more widespread effects. Here, we investigated the effect of acute systemic administration of the brain-penetrant COMT inhibitor tolcapone on tissue levels of dopamine, noradrenaline, and the dopamine metabolites 3,4-dihydroxyphenylacetic acid (DOPAC) and homovanillic acid (HVA). We examined PFC, striatum, hippocampus and cerebellum in the rat. We studied both males and females, given sexual dimorphisms in several aspects of COMT's function. Compared with vehicle, tolcapone significantly increased dopamine levels in the ventral hippocampus, but did not affect dopamine in other regions, nor noradrenaline in any region investigated. Tolcapone increased DOPAC and/or decreased HVA in all brain regions studied. Notably, several of the changes in DOPAC and HVA, particularly those in PFC, were more prominent in females than males. These data demonstrate that COMT alters ventral hippocampal dopamine levels, as well as regulating dopamine metabolism in all brain regions studied. They demonstrate that COMT is of significance beyond the PFC, consistent with its links with a broad range of behavioural phenotypes. Furthermore, they suggest that the impact of tolcapone may be greater in females than males, a finding which may be of clinical significance in terms of the efficacy and dosing of COMT inhibitors.

## Introduction

Catechol-O-methyltransferase (COMT) metabolises catechol-containing compounds, including dopamine [1], [2], [3], [4]. COMT inhibitors are used as an adjunctive treatment for Parkinson's disease, as they increase central L-DOPA availability [5]. Most COMT inhibitors have a limited ability to cross the blood-brain barrier; an exception is tolcapone, a brain-penetrant and specific COMT inhibitor [6], [7].

Given the use of COMT inhibitors for Parkinson's disease, the impact of tolcapone on dopamine levels has been well-studied in the striatum. In this region, COMT inhibition typically has little or no effect on dopamine levels, measured either in tissue homogenates or extracellularly [8], [9], [10]. These data are in keeping with the lack of a change in striatal dopamine levels in the COMT null mouse [2], [4], [11], [12]. In contrast, COMT inhibition increases dopaminergic transmission in the prefrontal cortex (PFC) [1], [3], consistent with findings of increased PFC dopamine levels in the COMT null mouse, compared with wild type littermates [2], [4].

In keeping with the importance of PFC dopamine for cognitive function [13], animal models show that lower COMT activity, mediated either pharmacologically [1], [3], [14] or genetically [15], results in better cognitive function. The human COMT gene contains a polymorphism (Val^158^Met) that influences enzyme activity. Although the evidence is somewhat inconsistent, there are numerous reports of associations between this polymorphism and cognitive function; similar to the findings in animals, it is the low activity Met^158^ allele that is associated with better performance [16], [17], [18]. Furthermore, Val^158^Met is robustly linked with the activation of the PFC (determined using functional magnetic resonance imaging; fMRI) during cognitive task performance [16], [19]. Studies of the impact of tolcapone on cognitive function in humans have reliably demonstrated interactive effects of drug treatment and Val^158^Met genotype [20], [21], [22], as would be predicted [13].

Although neurochemical, behavioural and neuroimaging data convergently implicate COMT in PFC function, emerging evidence suggests that its importance is not limited to this region, in line with its widespread expression [23]. For example, tolcapone improves hippocampal-dependent memory [24], extending neuroimaging data linking Val^158^Met genotype with hippocampal activation during emotional and cognitive processing [25], [26], [27]. Furthermore, although COMT clearly plays a lesser role in the striatum when compared with the PFC, an influential theory proposes that it may have subtle effects in the former region [28]. The hypothesis that COMT might impact on striatal function under certain circumstances (see Discussion) is attractive, given strong evidence linking Val^158^Met with striatal activation during reward processing (e.g. [29], reviewed in [30]), for which striatal dopamine is key [31], [32]. It is also consistent with interactive effects of tolcapone and Val^158^Met genotype on the performance of a reward task [20], similar to the relationship described above for working memory.

**Figure 1:**
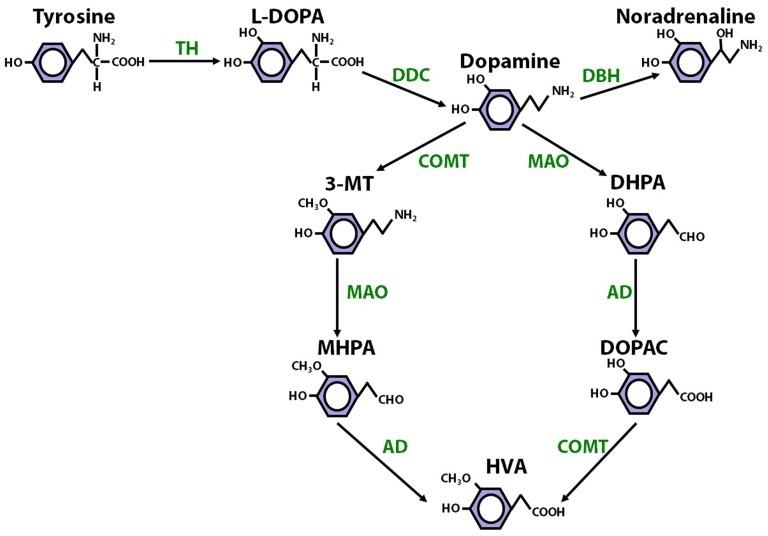
The synthesis and degradation of dopamine. Enzymes involved in the synthesis and catabolism of dopamine are highlighted in green. Dopamine is synthesised from tyrosine via L-DOPA (the rate-limiting step being the conversion of tyrosine to L-DOPA, mediated by tyrosine hydroxylase [TH]). Dopamine can be converted to noradrenaline, via the action of dopamine β-hydroxylase (DBH) or catabolised by the joint action of the enzymes COMT, monoamine oxidase (MAO) and aldehyde dehydrogenase (AH). Other abbreviations: dopamine decarboxylase (DDC); 3-methoxytyramine (3-MT); 3,4 dihydrophenylacetaldehyde (DHPA); 3-methoxy-4-hydroxyphenylacetaldehyde (MHPA).

Here, we aimed to investigate the impact of COMT in multiple brain regions. Therefore, we administered tolcapone (30 mg/kg i.p.) or vehicle to rats and measured tissue levels of dopamine and noradrenaline, and the dopamine metabolites 3,4-dihydroxyphenylacetic acid (DOPAC) and homovanillic acid (HVA), in the PFC, striatum, hippocampus and cerebellum (Figure 1). Notably, the majority of animal studies, and many human studies, (including our own) that have investigated aspects of COMT's function have focussed on male subjects, despite clear sexual dimorphisms in many aspects of COMT's function [33]. Therefore, we studied both male and female rats. Our findings confirm that COMT impacts on dopamine metabolism in multiple brain regions, and demonstrate a hitherto unappreciated role for regulating dopamine levels in the ventral hippocampus. Finally, they also suggest that the neurochemical effects of tolcapone may be more prominent in female rats, compared with males.

## Materials and Methods

### Ethics statement

All animal procedures were approved locally by the University of Oxford Committee on Animal Care and Ethical Review, and were carried out in accordance with the Animals (Scientific Procedures) Act 1986 and associated Home Office guidelines. All rats were handled in strict accordance with good animal practice as defined by the UK Home Office regulations.

### Animals and drugs

Age-matched (approximately 6–7 weeks) female (128–154 g) and male (202–230 g) Lister Hooded rats (Harlan-Olac, Bicester, UK) were housed in groups of three under standard conditions (lights on 7.30–19.30, 21±1°C temperature, 50% humidity, *ad libitum* food and water).

Tolcapone (Roche Products Ltd, Welwyn, UK), a specific COMT inhibitor [7], was suspended in vehicle (0.9% saline with a few drops of Tween-80) and administered intraperitoneally at a dose of 30 mg/kg. This dose significantly (∼70%) inhibits COMT activity [1].

### Tissue collection and sample preparation

Rats (n = 6 of each sex per drug group) were sacrificed by decapitation two hours after intraperitoneal drug or vehicle administration, since tolcapone's inhibition of central COMT is maximal at this time point [1]. PFC, striatum, dorsal and ventral hippocampus, and cerebellum were rapidly dissected on ice, snap frozen in isopentane and were stored at −80°C. On the day of HPLC analysis, tissue was thawed on ice and weighed. Tissue extracts were homogenised in 0.06 M perchloric acid (Sigma-Aldrich Company Ltd, Dorset, UK) for 10 s and centrifuged at 15000 rpm for 10 min. Catecholamines and metabolites were assayed in 50 µl samples of the supernatants. Results from dorsal hippocampus have previously been reported [24]; here we present ventral hippocampal results, although dorsal hippocampal results were included in the regional analyses, as described below.

### Measurement of tissue levels of catecholamines, HVA and DOPAC

Dopamine, noradrenaline, HVA and DOPAC were separated using a Microsorb C_18_ column (100×4.6 mm column; 3 µm C_18_ Microsorb particles; Varian Inc, Oxford, UK). The column was eluted isocratically with a degassed mobile phase consisting of 16% (v/v) methanol, 3 mM 1-Octanesulphonic acid (OSA), 1.07 mM EDTA and 0.12 M NaH_2_PO_4_.H_2_O, pH 3.3, at a flow rate of 1 ml/min. A glassy carbon electrode working at +0.7 V (vs. a Ag/AgCl reference electrode; BAS Instruments, Kenilworth, UK) was used for electrochemical detection (BAS LC-48 amperometric detector, BAS Instruments). Peak heights were converted to units of measurement by calibration against those elicited by the injection of standards containing 5 pmol of each substance of interest.

### Data analysis

Levels of catecholamines and metabolites are expressed as ng/g tissue. The effect of drug treatment and sex on catecholamines and metabolites was assessed separately for each region using analysis of variance (ANOVA), conducted using IBM SPSS Statistics version 19. Regional differences in catecholamine and metabolite levels, and their interactions with drug treatment and sex, were investigated using repeated-measures ANOVA, with region (striatum, PFC, dorsal hippocampus, ventral hippocampus and cerebellum) as the within-subjects factor. Greenhouse-Geisser corrections were applied where data failed Mauchley's test of sphericity (degrees of freedom are reported to one decimal place, rather than as integers, where this is the case). Data were not available for one male, saline, striatal sample and one female, saline, hippocampal sample, and so these cases were omitted from the regional analysis (as well as the relevant single-region analyses). Least significant difference (LSD) post hoc tests were used to explore significant interactions. The significance level was set at α = 0.05, with α = 0.1 considered a statistical trend.

## Results

### Effects of COMT inhibition and sex on catecholamines and metabolites

**Figure 2:**
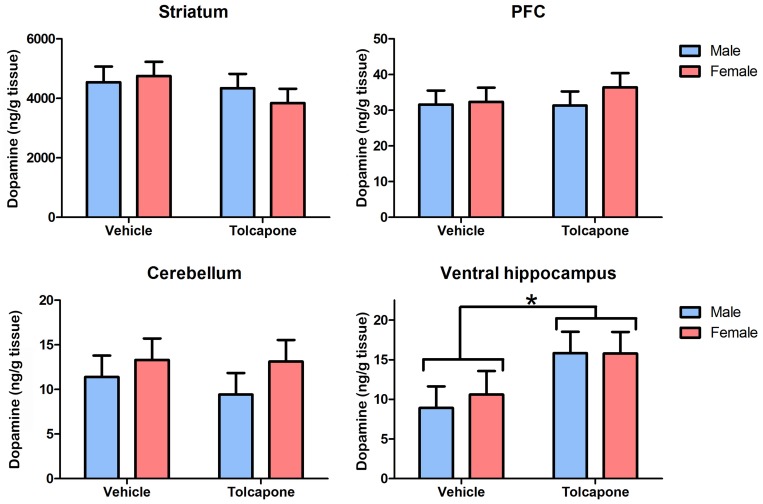
Tolcapone increased tissue dopamine levels in the rat hippocampus, but not other regions. Tissue levels of dopamine (expressed as ng/g tissue) are shown in the striatum, PFC, cerebellum and hippocampus. There were no main effects of sex, nor sex*treatment interactions. *p<0.05.

**Figure 3:**
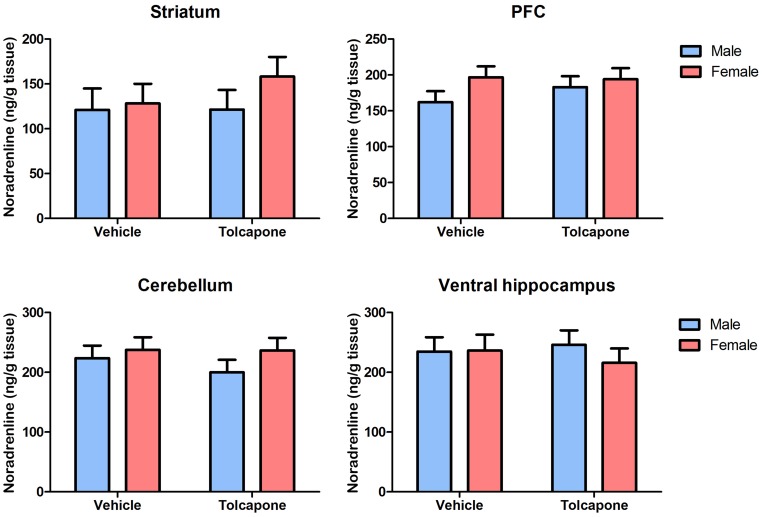
Tolcapone did not affect tissue noradrenaline levels in the rat brain. Tissue levels of noradrenaline (expressed as ng/g tissue) are shown in the striatum, PFC, cerebellum and hippocampus. There were no main effects of drug treatment or sex, nor sex*treatment interactions in any region.

Tolcapone treatment significantly increased dopamine levels in the ventral hippocampus, compared with the vehicle group (F_1,23_ = 4.7; p<0.05; Figure 2), in the absence of a main effect of sex or significant sex*drug treatment interaction (F's<1; *p*'s>0.1). There were no significant main effects on dopamine levels of drug treatment or sex, nor interactions between them, in the striatum, PFC or cerebellum (F's<1.36; *p*'s>0.1; Figure 2). There were no significant main effects of drug treatment or sex, nor an interaction between them, on noradrenaline levels in any of the regions examined (F's<1; *p*'s>0.1; Figure 3).

**Figure 4:**
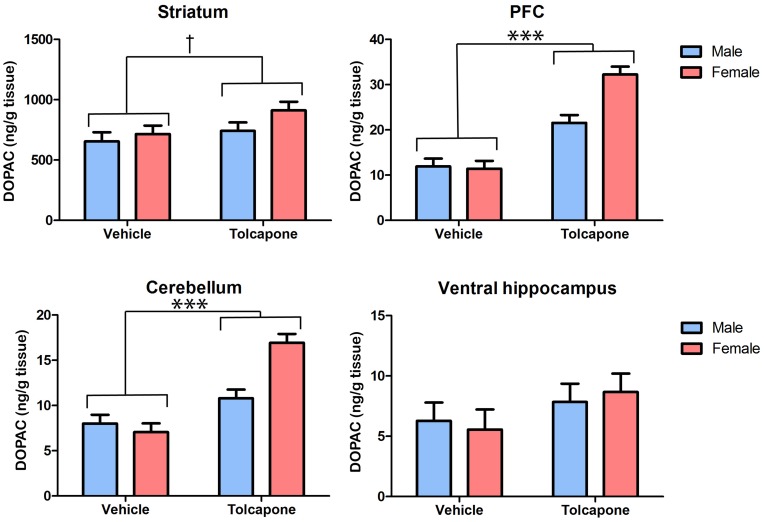
Tolcapone increased rat tissue DOPAC levels in all regions. Tissue levels of DOPAC (expressed as ng/g tissue) are shown in the striatum, PFC, cerebellum and hippocampus. For clarity, only main effects of drug treatment are shown. However, there were main effects of sex, and sex*treatment interactions, in the PFC and cerebellum (see text for details). ***p<0.0005.

Tolcapone treatment significantly increased DOPAC levels, compared to vehicle, in the PFC and cerebellum (F's>17.58; *p*'s<0.0005; Figure 4), and at trend level in the striatum (F_1,19_ = 3.90; *p*<0.1; Figure 4), but not in the ventral hippocampus (F_1,19_ = 2.3; p>0.1; Figure 4). In the PFC and cerebellum, there were significant sex*drug treatment interactions (F's>10.5; *p*'s<0.005; Figure 4). In both regions, sex differences (DOPAC higher in females than males) were found only in animals treated with tolcapone (*p*'s<0.0005); there were no sex difference in those given vehicle (*p*'s>0.1). However, in both regions, tolcapone significantly increased DOPAC irrespective of sex (*p*'s<0.005, except for males in the cerebellum, where this effect only reached trend level: *p*<0.1). The sex differences following tolcapone administration were large enough to result in main effects of sex on DOPAC levels (F's>7.1; *p*'s<0.005; female DOPAC>male DOPAC) in the PFC and cerebellum. There were no effects of sex, nor sex*drug treatment interactions, on this measure in the striatum or ventral hippocampus (F's<2.6; *p*'s>0.1).

**Figure 5:**
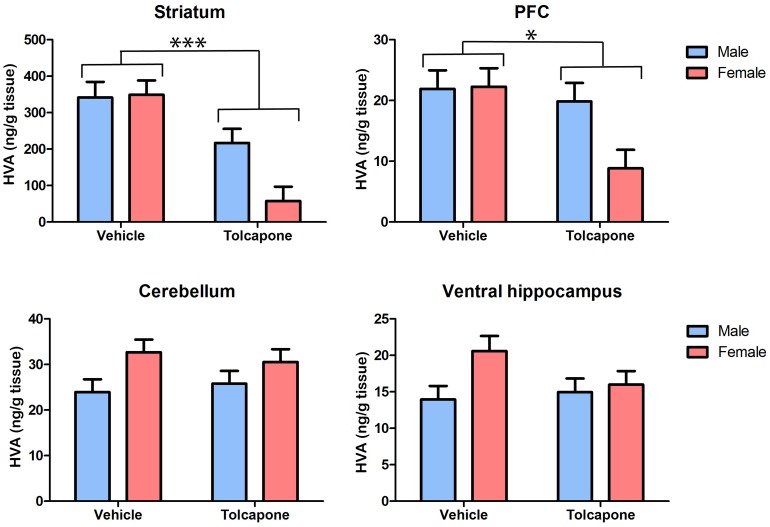
Tolcapone decreased tissue HVA levels in the rat striatum and PFC, but not the cerebellum and hippocampus. Tissue levels of HVA (expressed as ng/g tissue) are shown in the striatum, PFC, cerebellum and hippocampus. For clarity, only main effects of drug treatment are shown. However, there were trend effects of sex, and sex*treatment interactions, in the PFC and striatum (see text for details). *p<0.005; ***p<0.0005.

Tolcapone treatment significantly decreased HVA, compared with vehicle treatment, in the striatum and PFC (F's>6.49; *p*'s<0.05; Figure 5), but not in the cerebellum or ventral hippocampus (F's<1; *p*'s>0.1; Figure 5). In both the PFC and striatum there were trend level main effects of sex (F's>3.0; *p*'s<0.1), due to lower levels of HVA in females, compared with males. As was also the case for DOPAC, described above, this main effect of sex was driven by sex differences in the tolcapone- but not vehicle-treated animals. Thus, in both regions, there were trend-level sex*drug treatment interactions (F's>3.4; *p*'s<0.1) and post-hoc tests revealed significant sex differences (male HVA>female HVA) in tolcapone- (*p*'s<0.05) but not vehicle-treated (*p*'s>0.1) animals. In the cerebellum, there was a significant effect of sex on HVA levels, due to higher levels in females compared with males (F_1,20_ = 5.82; *p*<0.05), but this did not interact with drug treatment (F_1,20_<1; *p*>0.1). There were no significant main effects of sex, nor sex*drug treatment interactions in the ventral hippocampus (F's<2.4; *p*'s>0.1). Taken together with the dopamine and DOPAC findings outlined above, these results indicate that COMT inhibition alters dopamine metabolism in all regions studied. Intriguingly, they also suggest that, at least in some regions (explored further below), tolcapone's impact on dopamine metabolism may be greater in female than male animals.

### Regional differences in catecholamines and metabolites and their modulation by COMT inhibition and sex

There was a significant main effect of region on both noradrenaline and dopamine levels (F's>17.3; *p*'s<0.00005; Figures 2 and 3). For both catecholamines, post-hoc tests revealed that striatum and PFC differed significantly from all other regions, as well as from each other (*p*'s<0.01), but levels in subdivisions of the hippocampus and the cerebellum, did not differ from one another (*p*>0.1). Dopamine levels were highest in the striatum (4.3 mg/g tissue [mean of all animals]), intermediate in the PFC (32.3 ng/g tissue) and lowest in cerebellum (11.6 ng/g tissue) and hippocampus (dorsal: 10.8 ng/g tissue; ventral: 12.7 ng/g tissue). Noradrenaline levels were highest in the cerebellum (223.1 ng/g tissue) and hippocampus (dorsal: 221.8 ng/g tissue; ventral: 234.2 ng/g tissue), intermediate in the PFC (189.2 ng/g tissue) and lowest in the striatum (131.3 ng/g tissue). There were no significant interactions between region and treatment or sex (F's<1.7; *p*'s>0.1) in either case.

There was a main effect of region on DOPAC levels (F_1.0, 18.0_ = 432.1; *p*<0.00005), reflecting significant differences between all pairs of regions (*p*'s<0.005; Figure 4) except dorsal hippocampus and cerebellum (*p*>0.1). There was a trend-level region*sex interaction (F_1.0,18.0_ = 3.4; p<0.1), which arose due to there being no sex differences in ventral or dorsal hippocampus (F's<1; p's>0.1), but sex differences that were significant in PFC and cerebellum (p's<0.05), and present at trend level in the striatum (p<0.1). . There were no other significant interactions of region with either sex or drug treatment, nor their interaction.

There was a significant effect of region on HVA levels (F_1.0,18.3_ = 111.57; *p*<0.0005). Post-hoc tests revealed that this was due to significant differences between all pairs of regions (*p*'s<0.001), with the exception of PFC and ventral hippocampus, which were not significantly different (*p*>0.1), and PFC and dorsal hippocampus, which differed only at trend level (p's<0.1). There was a significant region*drug treatment interaction (F_1.0,18.3_ = 23.3; *p*<0.00005), due to effects of tolcapone on HVA levels in PFC and striatum (*p*'s<0.01) but not cerebellum or either hippocampal subregion (*p*'s>0.1). There was a trend-level interaction between region*sex (F_1.0,18.2_ = 4.3; *p*<0.1), which resulted from there being significant differences between all pairs of regions except PFC vs. ventral hippocampus in females (*p*'s<0.05), whilst in males HVA levels differed between all pairs of regions (*p*'s<0.05) except PFC vs. dorsal hippocampus (p>0.1). There was a trend-level sex*drug interaction (F_1,18_ = 3.6; *p*<0.1), resulting from sex differences being present in the tolcapone- (*p*<0.01) but not vehicle-treated (*p*>0.1) rats. Finally, there was a trend-level region*sex*drug treatment interaction (F_1.0,18.3_ = 3.3; *p*<0.1), which resulted from the sex differences in HVA levels in PFC and striatum occurring only after tolcapone treatment (*p*'s<0.05 for tolcapone group; *p*'s>0.1 for vehicle group), whilst trend level sex differences in the ventral hippocampus were present in vehicle- (*p*<0.1) but not tolcapone-treated (*p*>0.1) animals.

## Discussion

Our results demonstrate that acute COMT inhibition induces widespread alterations in catecholamine metabolism, as indexed by tissue levels of the dopamine metabolites DOPAC and HVA. Furthermore, COMT inhibition increased tissue dopamine concentrations within the ventral hippocampus, but did not alter noradrenaline in this (or any other) region. Intriguingly, some of these alterations were more prominent in female than male rats. Our findings suggest, consistent with other emerging findings [24], [27], [29], that COMT's significance for dopamine regulation is not limited to the PFC.

### COMT modulates catecholamine metabolism throughout the brain

COMT inhibition altered tissue levels of DOPAC and HVA in all brain regions studied except for ventral hippocampus, in keeping with its role in converting DOPAC to HVA (Figure 1). Even in the ventral hippocampus, there was a significant increase in dopamine, and a non-significant, numerical increase in DOPAC, in tolcapone- compared with vehicle-, treated animals. Therefore, COMT is present, active, and of significance for the metabolism of dopamine throughout the brain, consistent with its links with a diverse range of phenotypes [5], [18], [30], [34]. A number of studies have previously demonstrated effects of COMT, modulated both genetically and pharmacologically, on DOPAC and HVA levels in the PFC and striatum [1], [9], [11], [12]. However, the effects of COMT inhibition on dopamine function have been little-studied outside of these regions. The findings reported here are consistent with our earlier study that demonstrated an increase in dorsal hippocampal DOPAC and decrease in HVA after tolcapone administration [24]. To our knowledge, this study is the first to demonstrate a role for COMT in cerebellar dopamine metabolism.

Notably, whilst tolcapone increased DOPAC in all brain regions studied (albeit non-significantly in ventral hippocampus and striatum), its impact on HVA levels varied. Thus, whilst COMT inhibition reduced HVA in the PFC and striatum, it had no effect in the hippocampus or cerebellum. The mechanism underlying this differential effect is unclear; indeed information on dopamine function in these regions is generally sparse in comparison to the PFC and striatum. Since COMT activity is required for the formation of HVA, at least in the PFC and striatum [11], it seems unlikely that compensatory changes in other metabolic pathways underlie the lack of an effect of tolcapone in the cerebellum and hippocampus. Furthermore, the lack of a numerical difference between tolcapone and vehicle groups argues against the negative findings in hippocampus and cerebellum resulting merely from a lack of statistical power. It is plausible that the half-life of HVA is longer in these regions, for example as the result of regional differences in its clearance [35], and therefore that sustained differences in COMT activity are required to induce detectable changes. Furthemore, we have recently demonstrated that tolcapone decreases the extracellular, but not the total, pool of HVA in the dorsal hippocampus [24], suggesting that regional differences in the subcellular localisation of HVA and COMT (the precise distribution of which remains controversial [36], [37]) may also contribute.

### The relevance of COMT for catecholamine neurotransmission

We found that acute COMT inhibition increases levels of ventral hippocampal dopamine, but not noradrenaline, compared to vehicle. Given that hippocampal dopamine modulates memory performance [25], [38], [39], [40], our finding is broadly consistent with the beneficial effect of COMT inhibition on hippocampal-dependent memory performance [3], [24] and links between genetic variation in the human COMT gene and hippocampal activation [25], [26], [27]. However, there is a functional dissociation within the rodent hippocampus, with the dorsal hippocampus more important for spatial learning, and the ventral hippocampus prefentially involved in anxiety [41]. Thus, given that tolcapone increased ventral hippocampal dopamine, relative to vehicle, our data are arguably more directly relevant to reported links between COMT and anxiety, than those with memory (although, as outlined in the following section, a lack of an effect of COMT inhibition on tissue dorsal hippocampal dopamine does not exclude the possibility of functional effects of COMT in this region). Thus, they provide a possible mechanistic basis for the (albeit sexually dimorphic [33]) associations between the COMT Val^158^Met polymorphism and anxiety-related phenotypes [42], including obsessive compulsive disorder [43], as well as for reports of increased anxiety-related behaviours in the COMT null mice, compared with wild types [11], [15]. Notably, our findings provide a further example of specific effects of COMT on dopamine but not noradrenaline [1], [11], a result that is puzzling, given COMT's ability to metabolise both catecholamines. They are particularly striking in this case, given the ∼20 fold greater levels of noradrenaline, compared with dopamine, in the ventral hippocampus.

The absence of an effect of tolcapone on tissue dopamine levels in the PFC, striatum and cerebellum does not mean that COMT is not of significance for dopaminergic neurotransmission in these regions, although it does highlight the penetrance of the tolcapone-associated increase in ventral hippocampal dopamine. The assay used here measures the combined intracellular and extracellular neurotransmitter pools and therefore detects only relatively large and widespread neurochemical alterations. For example, even in the PFC, a region in which COMT has been consistently shown to regulate dopaminergic transmission [1], [2], [3], [4], its effect on basal tissue dopamine levels is rarely significant. Thus, although the initial study of the COMT null mouse demonstrated an increase in basal PFC tissue dopamine (which was limited to male mice) [11], a later study in the same transgenic mouse failed to replicate this finding [12]. Furthermore, a previous study also showed no effect of tolcapone administration on tissue levels of dopamine [9]. Even considering just the extracellular pool, COMT does not modulate basal dopamine levels in the PFC, but instead regulates dopaminergic transmission under conditions of increased dopamine release, as found during the performance of PFC-dependent tasks, for example [1]. Presumably, under baseline conditions, extracellular dopamine levels are regulated by uptake mechanisms, enzymatic degradation becoming significant only when dopamine release increases and the buffering capacity of the uptake mechanism is exceeded. Therefore, the relative importance of COMT for regulating dopamine levels in different brain regions is likely to be determined in large part by the availability and activity of local uptake mechanisms [18]. In turn, the relative scarcity of synaptic dopamine transporters in the PFC, compared with the striatum [44], [45] results in the greater significance of COMT in PFC compared with striatum. Evidence for the hypothesis that COMT's actions in the striatum are limited by uptake mechanisms comes from the demonstration that COMT inhibition potentiates the increase in striatal dopamine that occurs when the dopamine transporter is blocked [46]. We recently showed that Δ-9-tetrahydrocannabinol (THC) increases extracellular dopamine levels in the nucleus accumbens only when COMT is inhibited [47], providing further evidence that COMT can be of significance for subcortical dopamine under certain circumstances. In conclusion, although COMT undoubtedly plays a prominent role in modulating dopaminergic transmission in PFC, it will be of significant interest to assess the biological relevance of its role in subcortical regions, particularly given consistent links between COMT and striatal activation during reward processing (reviewed in [30]).

### Sexually dimorphic effects of tolcapone

A perhaps surprising aspect of our results is the prominent sex difference in the impact of tolcapone on dopamine metabolite levels. Specifically, females showed a greater tolcapone-related change in DOPAC in PFC and cerebellum (Figure 4), and in HVA in PFC and striatum (Figure 5). Given that these sexual dimorphisms were most prominent in animals given tolcapone, the most parsimonious explanation is that they are due to sex differences in the metabolism of tolcapone. To our knowledge, there is no published information concerning the effect of sex on these factors in the rat. However, a large study in patients with Parkinson's disease found no effect of sex on tolcapone clearance [48]. Furthermore, it is notable that there was no evidence for a sexually dimorphic effect of tolcapone on hippocampal DOPAC, suggesting some regional specificity for these sex effects. Therefore, it is possible that differential sensitivity to tolcapone is another sexually-dimorphic aspect of COMT's function [33], reflecting, for example, a sex difference in some aspect of COMT's expression or activity – albeit the lack of a sex difference in baseline tissue DOPAC or HVA suggests that any such effect must either be limited, or compensated for in some way. A baseline alteration in COMT function of this nature could contribute to its numerous sexual dimorphisms, including sex differences in its association with disease states such as obsessive compulsive disorder [33], [43]. Given the robust sexual dimorphisms in tolcapone's effect on PFC DOPAC and HVA, it would be of significant interest to compare the impact of tolcapone on cognitive function in male vs. female animals; to our knowledge, only male rats have been studied to date [1], [3]. The question could be of clinical relevance, given tolcapone's therapeutic potential as a treatment for cognitive dysfunction in a range of psychiatric disorders [20].

### Conclusions

We demonstrate that COMT is of significance for dopamine metabolism not just in the PFC but also in striatum, hippocampus and cerebellum. Furthermore, we provide the first direct evidence that COMT inhibition increases ventral hippocampal dopamine. Notably, the impact of tolcapone on dopamine metabolite levels was greater in female rats than males in the PFC, striatum and cerebellum. These findings highlight the hippocampus as another key region in which COMT may exert its effects on behaviour and brain function. The results are also of clinical significance, given the use of tolcapone as an adjunctive treatment for Parkinson's disease and its potential application as a treatment for cognitive dysfunction. It will be of interest to investigate to what extent clinically-relevant responses to tolcapone are sexually dimorphic.
